# Use of Internal Jugular Vein POCUS to Assess Intravascular Volume Status: A Study in Critically Ill Pediatric Post-Operative Cardiac Patients

**DOI:** 10.24908/pocusj.v10i02.18249

**Published:** 2025-11-17

**Authors:** Karunya Jayasimha, Wei Liu, William Hanna

**Affiliations:** Department of Pediatric Critical Care, Cleveland Clinic Children's, Cleveland, Ohio, USA

**Keywords:** Pediatrics, POCUS, Internal jugular vein, Volume status

## Abstract

**Background:**

Understanding the intravascular volume status in critically ill children is challenging. Central venous pressure (CVP) is commonly used but is invasive. Assessment of both the inferior vena cava (IVC) and the internal jugular vein (IJV) by point of care ultrasound (POCUS) have shown significant correlation with CVP in adults. Limited data exists in pediatric patients, especially with IJV measurements. This study aims to correlate IJV POCUS with CVP in mechanically ventilated post-operative children following congenital heart disease surgery.

**Methods:**

This prospective study was conducted in the pediatric cardiac critical care unit at a tertiary children's hospital. In addition to other variables, IJV/common carotid artery (CCA) ratio at inspiration and expiration was calculated using the largest diameters of both vessels. The IJV distensibility index (%) was calculated as the ratio of difference in maximal IJV diameter during inspiration and minimal IJV expiratory diameter to minimal IJV expiratory diameter × 100. CVP was obtained prior to POCUS measurements. The association between CVP and the IJV/CCA ratio, as well as IJV distensibility, were assessed using correlation coefficients and 95% confidence intervals.

**Results:**

Thirty-one patients were included, with a median age of 2 months (IQR [0, 6]) and median CVP value of 8.5 mm Hg (IQR 6-11). No significant correlations were found between CVP and IJV/CCA ratio (R= 0.030 p= 0.87) and IJV distensibility index (R= -0.19 p= 0.31).

**Conclusions:**

Although utilizing IJV POCUS to assess volume status may be advantageous given the limited access to IVC measurements in post-operative pediatric cardiac patients, our preliminary data suggests limited utility. Larger-scale studies are needed to establish a more definitive relationship between these variables.

## Introduction

Understanding the intravascular volume status of critically ill children to aid in appropriate hemodynamic management continues to be an ongoing clinical challenge at the bedside [1]. Of existing clinical tools, central venous pressure (CVP), despite its known limitations, continues to be regularly used as a surrogate marker of volume status [2]. However, measuring CVP requires placement and use of a central venous line, which is an invasive procedure associated with increased comorbidities during hospitalization [3]. Point of care ultrasound (POCUS) is a non-invasive tool that approximates CVP and has been utilized at the bedside for over four decades [4]. Inferior vena cava (IVC) diameter and collapsibility has been studied extensively in adults, though less so in pediatrics. There is a stronger positive correlation between IVC/aorta diameter ratio and CVP in children, and inconsistent correlations between IVC collapsibility index and CVP, in part related to scant normative age-specific data [5–9]. IVC distensibility index in relation to CVP has also been studied in adults and pediatrics with contradictory results [10–12]. More recently, adult literature has focused on internal jugular vein (IJV) parameters, which have the advantage of easier access to measure in post-operative patients with chest tubes and abdominal dressings that obscure the subxiphoid window. These studies have suggested that IJV diameter measurements increase significantly with increasing CVP [13,15]. Within pediatrics, there is currently no published data correlating IJV POCUS findings with CVP or other parameters of intravascular volume status.

This prospective cross-sectional study evaluated the correlation between common IJV POCUS measurements and CVP in pediatric patients with cardiac disease admitted postoperatively to the pediatric cardiac intensive care unit. This study is unique in existing literature for the following reasons: 1) Our population involves only critically ill post-operative cardiac children—a population at higher risk of hemodynamic deterioration in pediatric intensive care units, and therefore in better need of understanding intravascular volume status, and 2) Previous studies on this topic have been performed in adults but not children.

## Methods

This prospective cross-sectional study was performed between May 2023 to May 2024 at a tertiary care hospital after being approved by the Institutional Review Board. The primary aim was to investigate the correlation between IJV/common carotid artery (CCA) ratio and CVP. The secondary aim was to investigate a correlation between IJV distensibility index and CVP. Our inclusion criteria were postoperative, mechanically ventilated cardiac patients from 0-18 years with presence of an intrathoracic central line (internal jugular or right atrial line). Our exclusion criteria were clinically relevant tricuspid regurgitation, thrombosis of neck veins, and history of arrhythmia. For any patient meeting the above criteria, the guardian or parent of the patient was approached to obtain written informed consent while admitted into the pediatric cardiac critical care unit.

The POCUS examination and measurements were performed by two intensivists and conducted under the direct supervision of a senior intensivist certified in Critical Care Echocardiography by the National Board of Echocardiography and Critical Care Ultrasonography by the American College of Chest Physicians. Information taken from the study team was not shared with the clinical team, and thus did not influence any clinical decisions made by the clinical team.

The linear probe (L10-22) of the GE Venue fit model (Chicago, Illinois, USA) was used to obtain IJV/CCA ratio and distensibility index. The patients were placed in a supine position at the level of the bed with no pillow or other objects under their head. Ultrasound gel was applied over the linear probe and placed on the side of their neck contralateral to the intrathoracic central line. Slight manual pressure was used to compress the IJV to differentiate it from the CCA. The largest diameter of the CCA was obtained in the transverse view at the level of the cricoid cartilage. The largest diameter of the IJV was also obtained in the transverse anterior posterior axis view at the level of the cricoid cartilage at the end of inspiration and expiration. The IJV/CCA ratio was calculated at the end of inspiration and the end of expiration, assuming the largest diameter to be during expiratory phase and smallest during the inspiratory phase.

The technique for calculating distensibility index was similar to that described above. The anterior posterior diameter of the IJV was measured using the M-mode throughout the respiratory cycle. The IJV distensibility index (%) was calculated as the ratio of the difference in the maximal IJV anterior posterior diameter during inspiration and minimum IJV expiratory diameter to the minimum IJV expiratory diameter × 100.

The CVP was measured immediately before the sonographic examination. The bedside nurse measured it manually using a pressure manometer from the distal lumen of the central venous catheter. For this, the patient was placed supine and transducer appropriately zeroed (level of 4th intercostal space in the mid axillary line) prior to measurement. The POCUS users were blinded to the CVP readings to avoid bias.

SAS 9.4 software (SAS Institute, Cary, NC) was used to calculate sample size. Hossein-Nejad et al. found a strong correlation between the IJV/CCA ratio and CVP (r= 0.728–0.736) [15]. Assuming that the correlation was similar in our study (0.7), we calculated that a minimum of 43 patients would be needed in order to have 80% statistical power to detect a moderate association or above (> 0.4) at a significance level of 0.05.

Study data was collected and managed using REDCap electronic data capture tools hosted at Cleveland Clinic.

Normality of continuous variables was assessed by diagnostic plots and the Shapiro-Wilk test. Continuous variables were described using medians [25th, 75th percentiles] or mean ± standard deviation (SD); categorical variables were described using counts and percentages. Patients were stratified into two groups utilizing a CVP threshold of 7 mm Hg, which is a suggested marker for predicting volume responsiveness in prior existing adult studies focusing on IJV predictability [15,16]. Comparisons between CVP ≥ or < 7 mmHg categories were assessed using the Wilcoxon rank sum test or the pooled t-test for continuous variables, the Wilcoxon rank sum test for ordinal variables, and the Chi-square test or Fisher's exact test for categorical variables, as appropriate. The sign test was used to compare IJV measurements at inspiration and expiration within patients, with the mean of the paired differences evaluated by bias-corrected bootstrap confidence intervals (CIs). The univariate association between CVP with IJV/CCA ratio at inspiration, at expiration, and IJV distensibility were assessed using the Pearson correlation coefficients with 95% CIs. Log transformation was considered as needed. Receiver operating characteristics (ROC) analysis was performed using logistic regression models of CVP < 7 mm Hg or not for continuous IJV/CCA ratio at inspiration, at expiration, and IJV distensibility index, respectively; area under the ROC curves and corresponding 95% CIs were presented.

No missing values were observed in the data collected. All tests were two-tailed and performed at an overall significance level of 0.05. SAS 9.4 software (SAS Institute, Cary, NC) was used for all analyses and plots.

## Results

Thirty-one patients were included in the study and had a median age of 2 months (IQR [0, 6]). Of the patients, 38.7% were females. The median CVP value was 8.5 mm Hg (IQR 6-11). Patients with CVP < 7 mm Hg were less likely to be receiving an epinephrine infusion (50.0 vs. 95.2%, p= 0.007). The median positive end-expiratory pressure (PEEP) was 5.0 cm H2O [5.0-5.0] and the mean airway pressure was 8.4 cm H2O [7.8-9.0] with no significant differences between the two CVP groups. See Table 1 for additional demographic and clinical data.

**Table 1.** Demographic and clinical characteristics, overall and by central venous pressure (CVP) groups. PEEP= positive end-expiratory pressure

Factor	Total (N= 31)	≥ 7 mm Hg (N= 21)	< 7 mm Hg (N= 10)	p-value
Age (months)	2.0 [0, 6.0]	4.0 [0, 6.0]	2.0 [0, 6.0]	>.99
Sex				0.45
Male	19 (61.3)	14 (66.7)	5 (50.0)	
Female	12 (38.7)	7 (33.3)	5 (50.0)	
BMI	14.4 [13.2, 16.4]	14.7 [13.9, 16.7]	13.8 [12.2, 14.9]	0.18
Single/Biventricular Heart				0.22
Single Ventricle Heart	8 (25.8)	7 (33.3)	1 (10.0)	
Biventricular Heart	23 (74.2)	14 (66.7)	9 (90.0)	
Mean Arterial Pressure (mm Hg)	54.7 ± 9.6	53.4 ± 9.0	57.6 ± 10.8	0.26
Vasoactive Drugs	29 (93.5)	20 (95.2)	9 (90.0)	>.99
Type of Vasoactive: Epi	25 (80.6)	20 (95.2)	5 (50.0)	***0.007***
Type of Vasoactive: Mil	19 (61.3)	12 (57.1)	7 (70.0)	0.70
Type of Vasoactive: Vaso	2 (6.5)	2 (9.5)	0 (0)	>.99
Type of Vasoactive: Nipride	7 (22.6)	3 (14.3)	4 (40.0)	0.17
Mode				0.24
SIMV PRVC	25 (80.6)	17 (81.0)	8 (80.0)	
SIMV PC	3 (9.7)	1 (4.8)	2 (20.0)	
CPAP PS	3 (9.7)	3 (14.3)	0 (0)	
PEEP	5.0 [5.0, 5.0]	5.0 [5.0, 5.0]	5.0 [5.0, 5.0]	0.80
Mean Airway Pressure	8.4 [7.8, 9.0]	8.5 [8.0, 9.0]	8.0 [7.8, 9.0]	0.38

The mean IJV diameter was 0.45 cm [0.37, 0.57] at inspiration and the mean CCA diameter was 0.32 cm [0.25, 0.38]. The mean IJV/CCA ratio at inspiration was 1.5 ± 0.39, whereas the mean IJV distensibility index was 9.8 [7.0, 18.4].

**Figure 1.**
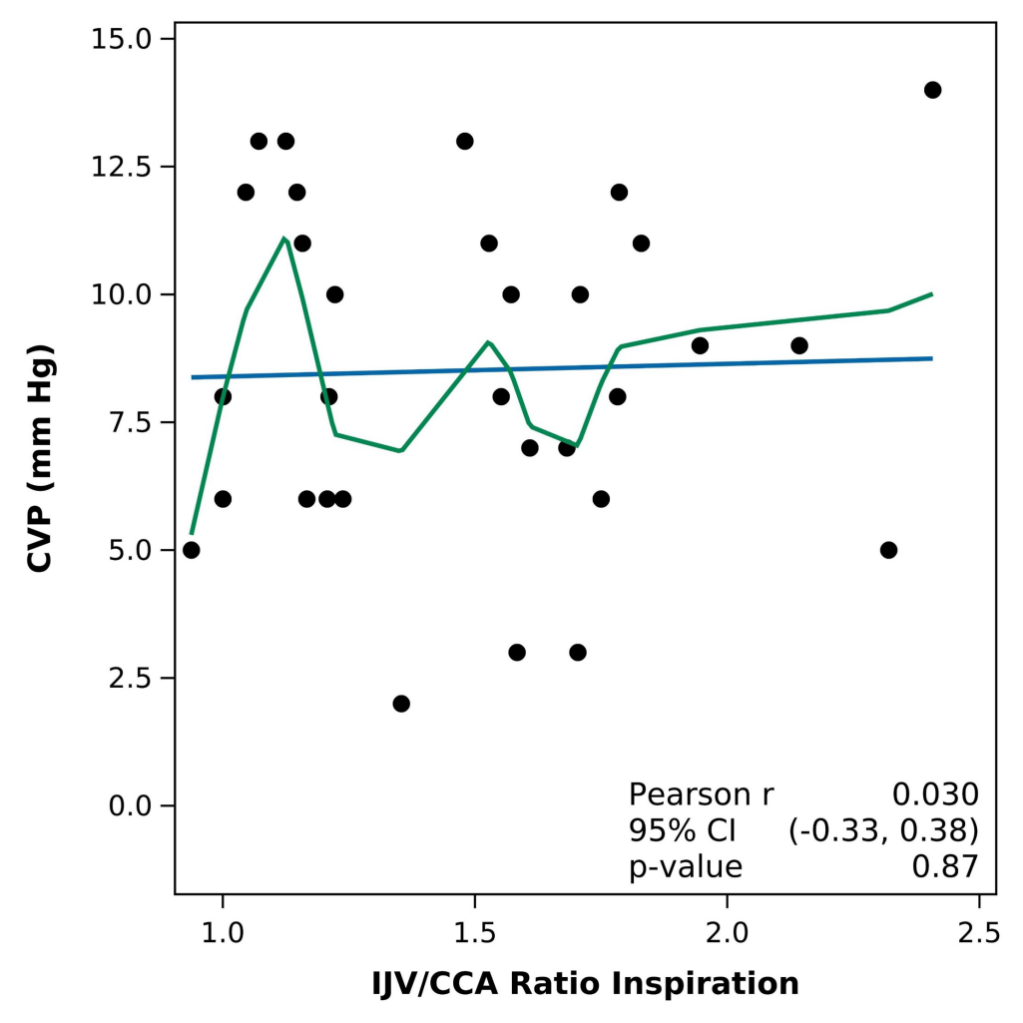
Pearson correlation of central venous pressure (CVP) with internal jugular vein (IJV)/common carotid artery (CCA) ratio (Blue line: fitted regression line; Green line: fitted loess). No significant correlation was found between the IJV/CCA ratio and CVP.

**Figure 2.**
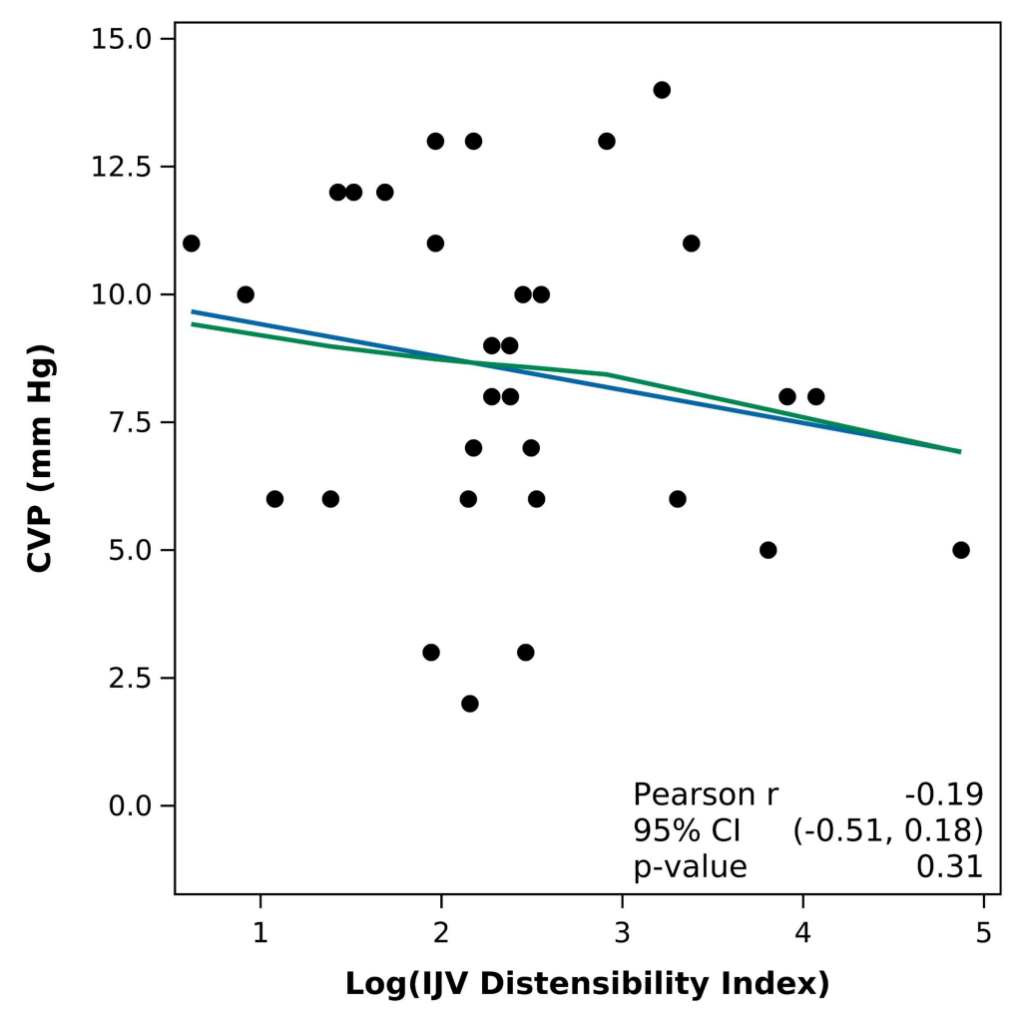
Pearson correlation of central venous pressure (CVP) with internal jugular vein (IJV) distensibility index (Blue line: fitted regression line; Green line: fitted loess. No significant correlation was found between the IJV distensibility index and CVP.

Furthermore, no significant correlations were found between the CVP and IJV/CCA ratio at inspiration (R= 0.030 p= 0.87) or IJV distensibility index (R= -0.19 p= 0.31). (Figures 1 and 2)

## Discussion

Our study sought to investigate the relationship between the IJV/CCA ratio and IJV distensibility index with CVP in mechanically ventilated postoperative pediatric cardiac patients within the cardiovascular intensive care unit. We found no significant correlation between IJV/CCA ratio and IJV distensibility index with CVP.

Among adult studies investigating IJV measurements, Hossein-Nejad et al. found a strong correlation between IJV/CCA ratio and CVP, exhibiting 90% sensitivity and 86% specificity in predicting a CVP < 10 cm H2O. However, their study was conducted in a cohort of spontaneously breathing patients [15]. To our knowledge, only two adult-based studies have been conducted investigating IJV measurements in predicting CVP in mechanically ventilated patients. Hilbert et al. discovered a moderate predictability of IJV measurements to identify patients with low (< 5 mm Hg) vs. high (>10 mm Hg) CVP in mechanically ventilated patients. However, they used the IJV ratio between 30-degree and 0-degree patient positions as their predictive measure [14]. Bano et al. observed a moderate correlation between the IJV/CCA diameter ratio and CVP (r= 0.401) in adult patients, however, that correlation becoming weak in the mechanically ventilated subgroup (r= 0.343), which mirrored our findings [13]. Given limited age/weight based normative data in pediatrics, more literature has focused on IVC-aortic diameter ratios. In these, the aorta is used as an internal control, as opposed to absolute venous measurements. With this approach, some studies suggest moderate predictability of IVC/aortic ratios in recognizing dehydration, albeit in spontaneously breathing children [16]. Very little evidence exists that specifically investigates IJV measurements in pediatrics. Elsadek et al. examined associations between the IJV measurements with the left ventricular end-diastolic area, which is a preload predictor akin to CVP, in mechanically ventilated pediatric cardiac patients, reporting a poor correlation (r= 0.2) [17].

The introduction of positive pressure ventilation (PPV) can complicate POCUS-based estimations of CVP through measurements of the great vessels. Lin et al. conducted a study to explore the impact of PPV on the IVC, observing that IVC/CCA ratio increased following the introduction of PPV [18]. This shift is believed to result from elevated intrathoracic pressure, which subsequently affects right atrial pressure and impedes venous return. Similarly, Han et al. demonstrated that the application of PEEP not only enlarged the size of the IJV but also minimized fluctuations in size caused by the respiratory cycle [19]. Within our study, no significant difference was found for mean airway pressure or PEEP when comparing CVP ≥ 7 vs. < 7 mm Hg groups (see Table 1). If differences in the CVP groups were found, it may suggest that PPV was a confounder in directly relating CVP to IJV measurements. The lack of a difference does not rule out this possibility. Also, of the vasoactives used, epinephrine was more often used in the CVP ≥ 7 group vs. the < 7 group. It has long been recognized that vasoactive medications such as epinephrine exert venoconstrictive effects and thus may positively influence venous return and increase CVP. However, larger studies may be needed to more effectively understand this finding and contextualize it with respect to POCUS-based measurements [20].

The bedside non-invasive assessment of intravascular volume status, particularly in pediatrics, remains challenging. Despite regular use in high-risk populations, CVP remains limited as an accurate predictor of intravascular volume status in isolation [21]. As an alternative, finding additional novel POCUS-based techniques and effective clinical outcome measures to assess their performance remains an area of ongoing research interest. The venous excess ultrasound score (VExUS) is a novel and potentially useful clinical tool that uses Doppler ultrasonography to grade the hepatic vein, portal vein, and intrarenal venous system as indicators of systemic venous congestion. It has shown a notable correlation with CVP and with clinical outcomes such as the development of acute kidney injury in adult patients [22]. Although one pediatric study found an association between elevated CVP and the intrarenal Doppler component of the VExUS score, the VExUS has not been effectively studied in the pediatric population and remains an opportunity for further research [23].

## Limitations

Our study has a range of limitations. Firstly, the findings may lack broader applicability since our study was conducted at a single center. Secondly, reaching definitive conclusions from the study regarding IJV-CVP correlations is limited due to the small sample size, which necessitates the need for larger studies. Thirdly, while all scans were performed by an intensivist trained in POCUS, the operator-dependent nature of sonographic measurements introduces potential variability, despite efforts to minimize it. Lastly, the exclusion of spontaneously breathing patients may have obscured potential correlations and precluded the investigation of how PPV influences IJV measurements.

## Conclusion

IJV measurements as a volume surrogate in pediatric post operative cardiac patients suggests limited utility in intubated patients. Larger-scale studies are needed to better define the relationship between these variables and to determine clinical applicability of findings, both in mechanically ventilated and spontaneously breathing populations.
